# Prospective per‐target analysis of the added value of the PrecisionPoint Transperineal Access System in cognitive prostate biopsy of MRI targets

**DOI:** 10.1002/bco2.462

**Published:** 2024-11-10

**Authors:** Luca Orecchia, Stefano Germani, Gaia Colalillo, Angelica Fasano, Matteo Ricci, Eleonora Rosato, Anastasios D. Asimakopoulos, Simone Albisinni, Enrico Finazzi Agrò, Guglielmo Manenti, Roberto Miano

**Affiliations:** ^1^ Urology Unit AOU Policlinico Tor Vergata University Hospital Rome Italy; ^2^ Department of Surgical Sciences University of Rome Tor Vergata Rome Italy; ^3^ Department of Diagnostic and Interventional Radiology, Molecular Imaging and Radiotherapy, AOU Policlinico Tor Vergata University of Rome Tor Vergata Rome Italy

**Keywords:** cognitive biopsy, local anaesthesia, PrecisionPoint, prostate biopsy, prostate biopsy complications, prostate cancer, prostate MRI, transperineal biopsy

## Abstract

**Objectives:**

The objective of this study is to evaluate the diagnostic performance of perineal access cannulas tethered to a biplanar ultrasound probe in cognitive transperineal prostate biopsies of targets identified by multiparametric magnetic resonance imaging (mpMRI) by comparing the results of the PrecisionPoint (PP) Transperineal Access System with the double‐freehand (DFH) technique.

**Patients and methods:**

All patients who underwent cognitive transperineal prostate biopsy of mpMRI targets using the PP or DFH technique between November 2020 and September 2023 were enrolled. All data related to mpMRI target biopsies were stratified by technique, visibility in transrectal ultrasound and analysed by comparing PP versus DFH. A standardised anaesthesia protocol with 1% mepivacaine was used in all biopsies. The tolerability of the procedures was assessed using a visual analogue scale (VAS).

**Results:**

The number of mpMRI targets sampled was 166 in PP and 242 in DFH. In target biopsies, the PP system was associated with better diagnostic performance for clinically significant prostate cancer (Gleason score ≥3 + 4) compared to DFH for both ultrasound‐visible targets (61.4% vs. 48.0%) and non‐visible targets (41.4% vs. 14.9%) (*p* = 0.02). A higher rate of positive cores was obtained from targets sampled with PP (57.7% vs. 49.6%, *p* = 0.0002). The PP system was associated with the retrieval of significantly longer cores (*p* < 0.0001). There was no significant difference between the techniques regarding pain reported during the biopsy, with a median VAS of 2.7/10, although the PP device required a lower amount of anaesthetic in the periprostatic planes (4.3 ± 2.0 mL vs. 5.9 ± 1.9 mL, *p* < 0.0001).

**Conclusion:**

The PrecisionPoint Transperineal Access System enabled more precise and higher quality biopsies, resulting in improved histological characterisation of prostate cancer compared to the DFH approach. The use of a perineal cannula did not increase the pain perceived by patients and also required less local anaesthetic during the biopsy.

## 1 INTRODUCTION

**FIGURE 1:**
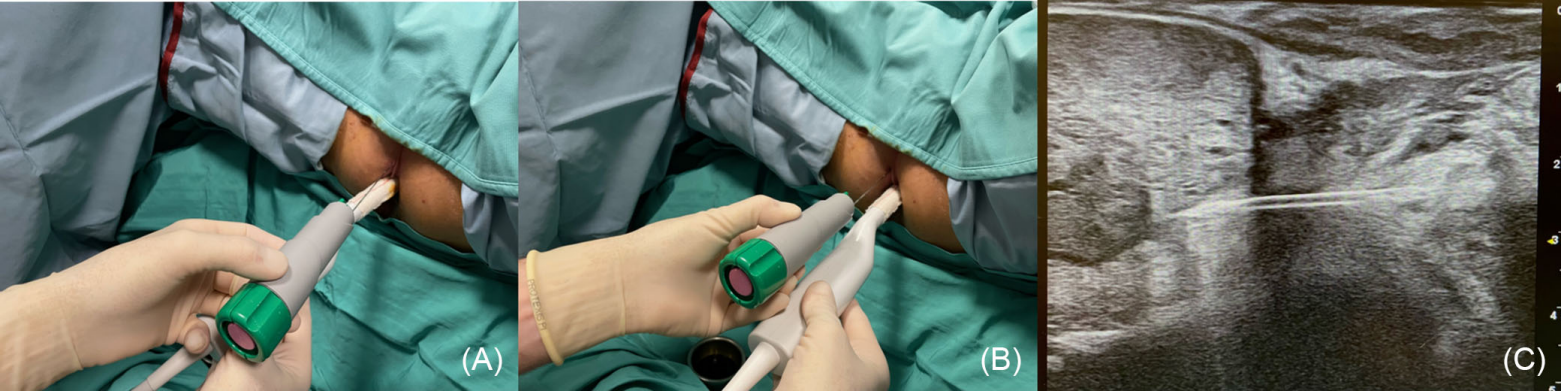
Double‐freehand transperineal prostate biopsy technique. (A) Inadequate needle alignment to the ultrasound probe and needle tract distortion. (B) Adequate needle alignment to the ultrasound probe, with no distortion. (C) Ultrasound view of an appropriately aligned needle in double‐freehand transperineal prostate biopsy.

The adoption of the transperineal approach to prostate biopsy (TP‐Bx) is gaining increasing interest due to mounting criticism towards the use of the transrectal route.^1^ Although first described in 1922, TP‐Bx has historically struggled to gain the same acceptance as the transrectal route due to lower tolerability, higher technical difficulty and greater resource requirements.^2^ In the modern era, the standard procedural approach involved using a brachytherapy grid mounted on a stepper unit. However, this method was associated with high post‐procedural complication rates and the necessity of general anaesthesia due to poor patient tolerability.^3^ Consequently, less invasive and more sustainable transperineal biopsy techniques were developed.^4^, ^5^, ^6^ The double‐freehand (DFH) technique with cognitive registration and local anaesthesia represents the least resource‐intensive TP‐Bx modality, despite its complexity due to the need for constant alignment of the biopsy needle with the ultrasound beam from the transrectal probe^7^ (Figure 1). To provide less complex alternatives to DFH and less invasive options than general anaesthesia with a brachytherapy grid, various devices have been developed, including perineal access cannulas. Since its first description by Novella et al. in 2003, the use of a coaxial cannula untethered to the ultrasound probe has been a potential addition to the DFH technique.^8^ The CamPROBE (The CAMbridge PROstate Biopsy DevicE, Cambridge, UK) cannula also employs this concept.^9^ These devices aim to minimise the number of perineal punctures, thereby increasing procedural tolerability while stabilising the needle tract and reducing needle distortion. However, they do not eliminate the complexity of needle alignment inherent in DFH techniques. To this purpose, the recently introduced PrecisionPoint (PP) Transperineal Access System (Perineologic, Cumberland, MD, USA), integrates with the ultrasound probe to simplify the procedure, provide adequate tolerability and minimise perineal punctures.^10^ Concurrently, several techniques for local anaesthesia in TP‐Bx have been described,^11^ and no consensus on this aspect has been reached.

Contrarily to international trends, the reference biopsy approach at the Urology Unit at the University of Rome Tor Vergata has been DFH TP‐Bx since the early 1990s. In May 2021, the PP system was added to the available equipment. Following the establishment of an internal pathway for pre‐biopsy multiparametric magnetic resonance imaging (mpMRI), our centre initiated a prospective audit in November 2020 to perform a granular analysis of all prostate biopsy outcomes. As DFH technique had been the established approach for over two decades, this study aims to analyse audited data to investigate the impact of introducing the PP system on the clinically significant prostate cancer (csPCa) diagnosis rates and reported procedural tolerability at our centre.

## 2 PATIENTS AND METHODS

### 2.1 Prostate biopsy pathway

Indication to perform prostate biopsy was given during outpatient counselling, considering health status, hereditary factors, digital rectal examination (DRE) results, prostate specific antigen (PSA) values, PSA‐density (PSA‐d) and pre‐biopsy mpMRI results. mpMRIs were either performed internally, on patients pre‐screened by the local PCa Working Oncology Group and scanned on a 3 T machine according to PI‐RADS v2.1 standards, or obtained from external centres. Internal mpMRIs, reported by expert uro‐radiologists,^12^, ^13^, ^14^ achieved a PI‐QUAL v1 score of 5/5 upon external evaluation.^15^ Patients with PSA levels above 50 ng/mL and a strong clinical suspicion of advanced disease were exempted from mpMRI. Each scan was then reviewed by a highly experienced urologist alongside training urologists responsible for performing the biopsy. Each urologist at our centre was accredited for mpMRI reading by successfully completing the MRI PRO course (MRI PRO Pty Ltd, Victoria, Australia).^16^ After image revision, patients underwent a pre‐biopsy planning transrectal ultrasound (TRUS) using a Esaote MyLab™X8 ultrasound platform (Esaote S.p.A., Genova, Italy) mounting a 3–13 MHz biplanar transrectal probe to identify and document hypoechoic areas corresponding to suspicious mpMRI targets, when visible. During the biopsy planning phase, sectors harbouring secondary and tertiary PI‐RADS 3 targets or tertiary PI‐RADS 4 were allocated to systematic sampling only, to minimise potential biopsy‐related morbidity associated with collecting a high overall number of targeted cores. Similarly, some high‐volume PI‐RADS 5 targets were included in a systematic sampling strategy when their targeting coincided with the systematic sampling of the prostate gland.

### 2.2 Operative procedures

TP‐Bx was conducted in an ambulatory outpatient setting without an anaesthetist. Selected patients with severe cardiovascular comorbidities or anal stenosis could receive the biopsy as day surgery for enhanced anaesthesia monitoring. A standardised safety protocol required blood tests and urine culture within 15 days before the biopsy. Patients with positive urine cultures were scheduled for biopsy after completing an antibiotic course and resolving the infection. Between November 2020 and December 2022, all patients received antibiotic prophylaxis with a single intra‐procedural infusion of 2 g of cefazolin. Subsequently, following the publication of NORAPP trial,^17^ the intra‐procedural administration of antibiotics was limited to cases considered at high risk for complicated urinary tract infections. At the end of each biopsy, patients rated intra‐procedural pain using a visual analogue scale (VAS) and were observed until spontaneous micturition was obtained.

### 2.3 Standardised technique and training

**FIGURE 2:**
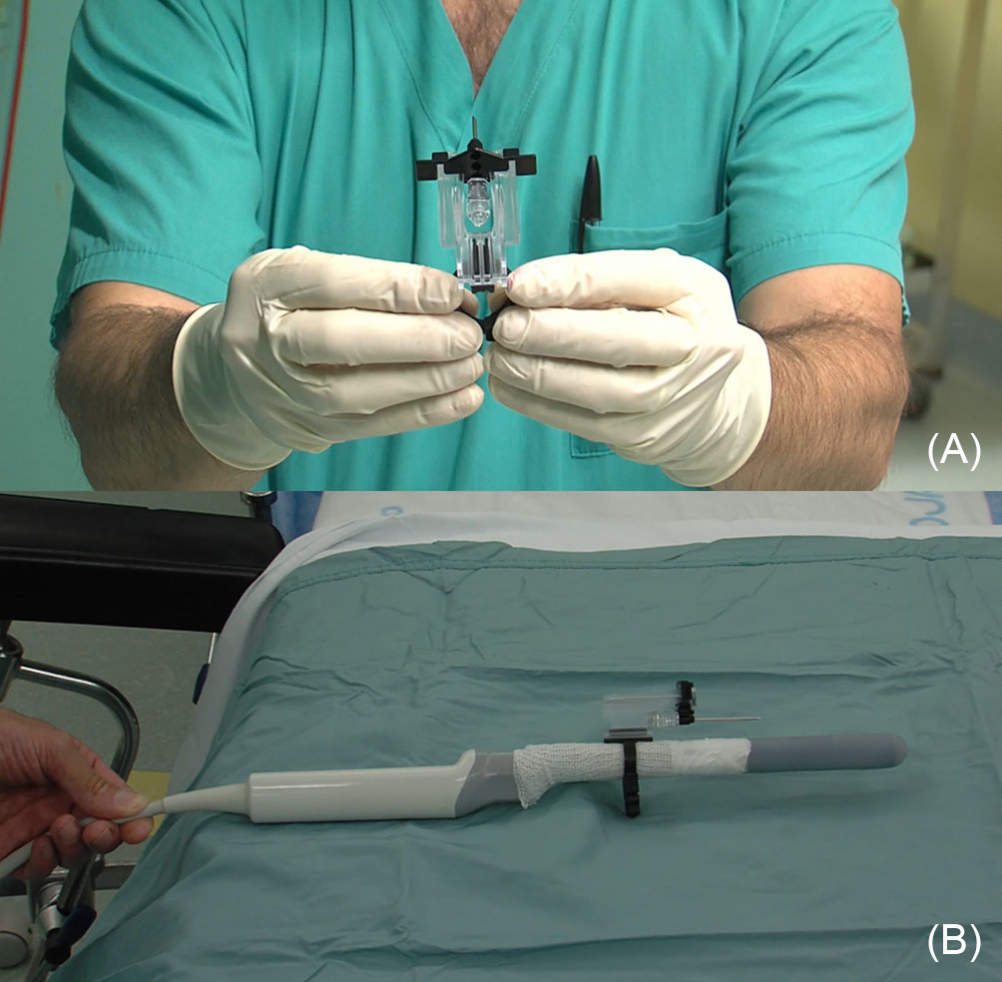
(A) Frontal view of the three components of the PrecisionPoint Transperineal Access System after assembly, with the 15 G perineal access cannula inserted into the lowermost of the apertures of the needle carriage. (B) Tethering of the device to a biplanar transrectal ultrasound probe; proper positioning ensures that the tip of the 15 G cannula intercepts the part of the beam emitted by the linear transducer that is closest to the operator. Padding made from cohesive bandage ensures adequate compatibility of the clamp/rail subassembly with probes of non‐circular cross‐sections.

The biopsies were performed by training urologists under supervision by an expert tutor, with occasional procedures performed by urology specialists. Patients were positioned in lithotomy and the perineum disinfected with iodine solution. A two‐step local anaesthesia protocol with 1% mepivacaine was used. Using a 23 G needle, a portion of anaesthetic was infiltrated on each side in the subcutaneous perineal area approximately 2–3 cm above the anal sphincter at the 2 and 10 o'clock positions. Subsequently, using a 22 G spinal needle under TRUS guidance, an additional portion of anaesthetic was instilled on each side both in the Denonvilliers' space near the prostatic neurovascular plexus and in the para‐apical region, instilling the drug between the prostatic capsule and the medial surface of the endopelvic fascia, combining the ‘peri‐prostatic’ and ‘apical’ approaches.^11^, ^18^, ^19^ Biopsies were performed using systematic, targeted and systematic, or only targeted strategies based on individual case characteristics. A single‐use 18 G/20 cm cutting needle with a penetration depth of 22 mm was employed. To minimise histological grade underestimation, a minimum of three samples were taken from each target area, with at least an additional core collected adjacent to the target for perilesional sampling.^20^, ^21^ Targeted sampling preceded systematic sampling in every biopsy. Systematic biopsies were collected from the peripheral zone according to a local zonal scheme, with at least two cores taken from each sector, avoiding biopsy repetition in mpMRI target areas. Training urologists learnt the procedure using both DFH and PP technique, initially focusing on biopsies with the PP to supersede the need to manually align the needle with the ultrasound beam (Figure 2). Subsequently, they underwent progressive training, starting with systematic biopsies using the DFH technique and advancing to mpMRI‐targeted biopsies. This modular training curriculum was designed to minimise reduction of accuracy in targeted biopsy during training; patient allocation to DFH or PP technique was regulated by the supervising expert tutor according to trainees' progress within curriculum. Most biopsies were done with cognitive registration, with MRI/TRUS software fusion being performed in selected cases such as PI‐RADS 3–4 lesions in challenging positions. The review and reporting of the collected materials were performed according to the 2019 International Society of Urological Pathology (ISUP) Consensus Conference on Gleason grading of prostate carcinoma.^22^

### 2.4 Definition of the study population and analysed data

This study aimed to evaluate the diagnostic performance of the PP device in biopsies performed with cognitive registration compared to the historically used DFH technique. To this end, the results of mpMRI‐target sampling were identified as the ideal indicator to assess any additional value of the device. Given the variability in indications and biopsy techniques, specific exclusion criteria were established. Patients were excluded if they were biopsied:

- Without a pre‐biopsy mpMRI;
- Using a systematic sampling strategy in the context of mpMRI PI‐RADS 1–2;
- Using exclusively a systematic sampling strategy, omitting targeted sampling despite having mpMRI targets PI‐RADS ≥3;
- Using MRI/TRUS software fusion registration.

**FIGURE 3:**
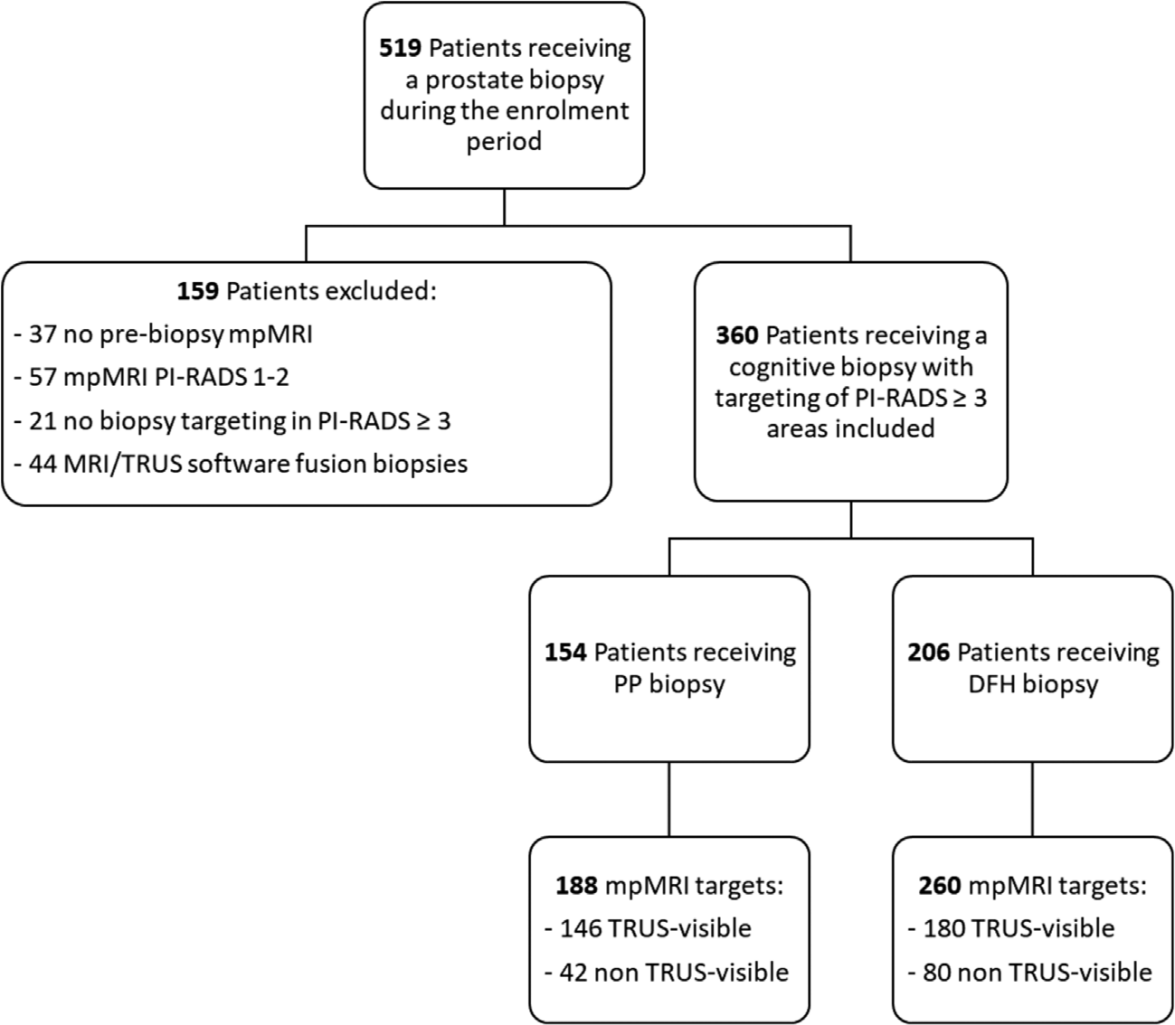
Summary of the inclusion, exclusion and stratification criteria for the study population. DFH, double‐freehand; mpMRI, multiparametric magnetic resonance imaging; PP, PrecisionPoint; TRUS, transrectal ultrasound.

The included patients were stratified by biopsy technique: PP and DFH. This study analysed results per individual target rather than per patient. For patients with multiple mpMRI targets, sampling results of each target were documented and analysed separately. Further stratification was based on the visibility of mpMRI targets on the planning TRUS. Exclusion and stratification criteria are summarised in Figure 3. The enrolment period extended from the start of prospective monitoring on November 2020 to the last biopsy session on September 2023. Subsequent enrolment was censored to avoid missing data from the most recent biopsies. Data collection and analysis were conducted prospectively using a dynamic data‐based audit and quality control system to monitor all service indicators adequately. Clinical data were entered into a computerised database updated monthly, with descriptive analyses automatically conducted using programmed analysis sheets. Analysed variables included demographics, home medical therapy, DRE results, serum PSA level, mpMRI results, visibility of targets on the planning TRUS, antibiotic prophylaxis, anaesthesia protocol, postoperative VAS pain assessment, number of cores taken for each target, length and degree of fragmentation of collected cores and histological examination outcomes. The analysis of mpMRI‐targeted biopsy results was the primary objective of the study and focused on three variables: the percentage of diagnosed clinically indolent (ci) PCa and csPCa, the positivity rate of cores for PCa and the dimensional and fragmentation analysis of the cores. Secondary objectives included assessing results for targets visible or not visible on the planning TRUS and evaluating the tolerability of the biopsy procedure between PP and DFH.

### 2.5 Statistical analysis

Continuous variables were described by median and interquartile range (IQR), tested for normality with the Kolmogorov–Smirnov test, then analysed with a two‐sample, unpaired *t*‐test or Wilcoxon rank‐sum test when appropriate. Categorical variables were presented as absolute frequencies and percentages, tested with *χ* ^2^ test or Fisher–Yates test according to sample size. Statistical significance was set at *p* < 0.05. Descriptive statistics were automatically executed by worksheets coded in Microsoft Excel (Microsoft Co., Redmond, WA, USA). All statistical analyses were performed on SPSS v28 (IBM, Armonk, NY, USA).

## 3 RESULTS

### 3.1 Demographics and clinical baseline data

The study cohort included a total of 360 patients, with 154 undergoing biopsy using the PP technique and 206 with the DFH technique, with an average age of 69 years at the time of biopsy. No statistically significant differences were observed in the distribution of baseline demographic and clinical variables, including DRE results (Table 1). A total of 37 patients under active surveillance for ISUP 1 acinar adenocarcinoma who underwent restaging biopsy were included, and 51.0% of the pre‐biopsy mpMRIs were performed at our centre.

**TABLE 1:** Demographics and clinical baseline data.

	PP group (*n* = 154)	DFH group (*n* = 206)	*p*‐Value
Median (IQR), average (SD) or *n* (%)			
Age at biopsy (years)	69.2 (±7.7)	69.0 (±8.0)	ns
Biopsy history			
First biopsy	119 (77.3%)	160 (77.7%)	
Previous negative biopsy	20 (13.0%)	24 (11.7%)	ns
Active surveillance	15 (9.7%)	22 (10.7%)	
Positive DRE	87 (56.5%)	110 (53.4%)	ns
PSA (ng/mL)	7.80 (5.23; 10.77)	7.63 (4.88; 10.65)	ns
Prostate volume (mL)	47 (34; 62)	50 (37; 67)	ns
PSA‐d (ng/mL/cc)	0.17 (0.11; 0.26)	0.15 (0.10; 0.23)	ns
mpMRI PI‐RADS			
PI‐RADS 3	18 (11.7%)	36 (17.6%)	
PI‐RADS 4	77 (50.0%)	89 (43.7%)	ns
PI‐RADS 5	59 (38.3%)	81 (39.7%)	

Abbreviations: DFH, double‐freehand; DRE, digital rectal examination; IQR, interquartile range; ns, not significant; PP, PrecisionPoint; PSA, prostate specific antigen; PSA‐d, PSA‐density.

The median PSA‐d value observed was 0.17 ng/mL/cc for the PP group and 0.15 ng/mL/cc for the DFH group, with the first quartile of the variable distribution ≥0.10 ng/mL/cc in both groups. The distribution of PI‐RADS 5 mpMRIs was homogeneous between the two groups, while a lower percentage of PI‐RADS 4 mpMRIs was observed in the DFH group (43.7% vs. 50.0%).

A notable 96.1% of the biopsy procedures were performed by urologists in training under supervised autonomy, with 91.4% of the cases conducted in an outpatient ambulatory setting.

### 3.2 Analysis of mpMRI targets

The number of reported mpMRI targets was 188 for the PP group and 260 for the DFH group, corresponding to 1.22 targets per patient in the PP group and 1.26 in the DFH group. There was no statistically significant difference in the distribution of targets according to PI‐RADS, despite a higher frequency of PI‐RADS 4 in both groups.

A significant difference in the visibility of PI‐RADS 3 targets on staging TRUS was observed in the PP group. However, statistical significance was not reached for PI‐RADS scores 4 and 5, although both were more frequently visible in the PP group (Table 2).

**TABLE 2:** Analysis of multiparametric magnetic resonance imaging (mpMRI) targets.

	PP group targets (*n* = 188)	DFH group targets (*n* = 260)	*p*‐Value
Median (IQR), average (SD) or *n* (%)			
mpMRI targets per patient	1.22	1.26	ns
mpMRI target PI‐RADS			
PI‐RADS 3	24 (12.7%)	55 (21.1%)	
PI‐RADS 4	100 (53.1%)	122 (47.0%)	ns
PI‐RADS 5	64 (34.0%)	83 (31.9%)	
Target visibility on TRUS	146 (76.4%)	180 (69.2%)	ns
PI‐RADS 3	17 (70.0%)	25 (46.0%)	0.03
PI‐RADS 4	73 (73.0%)	84 (68.9%)	ns
PI‐RADS 5	56 (87.5%)	71 (85.5%)	ns
Target maximum diameter (mm)	11.0 (6.0; 15.0)	10.0 (6.0; 15.0)	ns

Abbreviations: DFH, double‐freehand; IQR, interquartile range; ns, not significant; PP, PrecisionPoint; TRUS, transrectal ultrasound.

### 3.3 Analysis of mpMRI‐targeted biopsies

During the biopsy process, not all mpMRI targets were subjected to targeted biopsy as per biopsy planning. Specifically, targeted biopsy was omitted for 22 targets in the PP group and 18 in the DFH group. The distribution of targets not subjected to selective biopsy in PP and DFH was respectively as follows: PI‐RADS 3 *n* = 5 (20.8%), PI‐RADS 4 *n* = 13 (13.0%) and PI‐RADS 5 *n* = 4 (6.3%) for PP, and PI‐RADS 3 *n* = 8 (14.5%), PI‐RADS 4 *n* = 7 (5.7%) and PI‐RADS 5 *n* = 3 (3.6%) for DFH.

Consequently, a target‐based analysis was conducted on 166 targeted biopsies performed using the PP technique and 242 using DFH (Table 3). The use of the PP device was associated with a higher rate of csPCa diagnosis for targets visible on TRUS compared to DFH (61.4% vs. 48.0%) and a lower rate of ciPCa diagnosis (15.3% in PP vs. 28.0% in DFH), while maintaining a stable percentage of negative targeted biopsies (*p* = 0.02). A higher percentage of csPCa diagnoses was observed across all PI‐RADS stratifications, reaching statistical significance for PI‐RADS 4 targets, with 55.1% of csPCa diagnosed in PP compared to 39.0% in DFH (*p* = 0.04). A similar finding was observed for biopsies from targets not visible on TRUS, with a significantly higher csPCa diagnosis rate in PP (41.4% vs. 14.9%, *p* = 0.02). A significantly lower rate of negative targeted biopsies was also observed for non‐visible targets (41.4% PP vs. 62.7% DFH). This result was strongly influenced by the sampling of non‐visible PI‐RADS 4 targets, where the distribution difference of results reached the lower limit of statistical significance (*p* = 0.045).

**TABLE 3:** Diagnostic performance of multiparametric magnetic resonance imaging (mpMRI)‐targeted biopsies.

	PP group targets (*n* = 166)			DFH group targets (*n* = 242)			*p*‐Value
	Negative	ciPCa	csPCa	Negative	ciPCa	csPCa	
*n* (%)							
TRUS‐visible mpMRI targets							
PI‐RADS 3	7 (50.0%)	3 (21.4%)	4 (28.6%)	16 (64.0%)	6 (24.0%)	3 (12.0%)	ns
PI‐RADS 4	17 (24.6%)	14 (20.3%)	38 (55.1%)	18 (22.0%)	32 (39.0%)	32 (39.0%)	0.04
PI‐RADS 5	8 (14.8%)	4 (7.4%)	42 (77.8%)	8 (11.8%)	11 (16.2%)	49 (72.1%)	ns
Total	32 (23.3%)	21 (15.3%)	84 (61.4%)	42 (24.0%)	49 (28.0%)	84 (48.0%)	0.02
On TRUS‐visible mpMRI targets							
PI‐RADS 3	3 (60.0%)	1 (20.0%)	1 (20.0%)	17 (77.3%)	5 (22.7%)	0	ns
PI‐RADS 4	8 (44.4%)	2 (11.1%)	8 (44.4%)	22 (66.7%)	7 (21.2%)	4 (12.1%)	0.045
PI‐RADS 5	1 (16.7%)	2 (33.3%)	3 (50.0%)	3 (25.0%)	3 (25.0%)	6 (50.0%)	ns
Total	12 (41.4%)	5 (17.2%)	12 (41.4%)	42 (62.7%)	15 (22.4%)	10 (14.9%)	0.02

Abbreviations: ciPCa, clinically indolent prostate cancer (Gleason score = 3 + 3); csPCa, clinically significant prostate cancer (Gleason score ≥ 3 + 4); DFH, double‐freehand; ns, not significant; PP, PrecisionPoint; TRUS, transrectal ultrasound.

**FIGURE 4:**
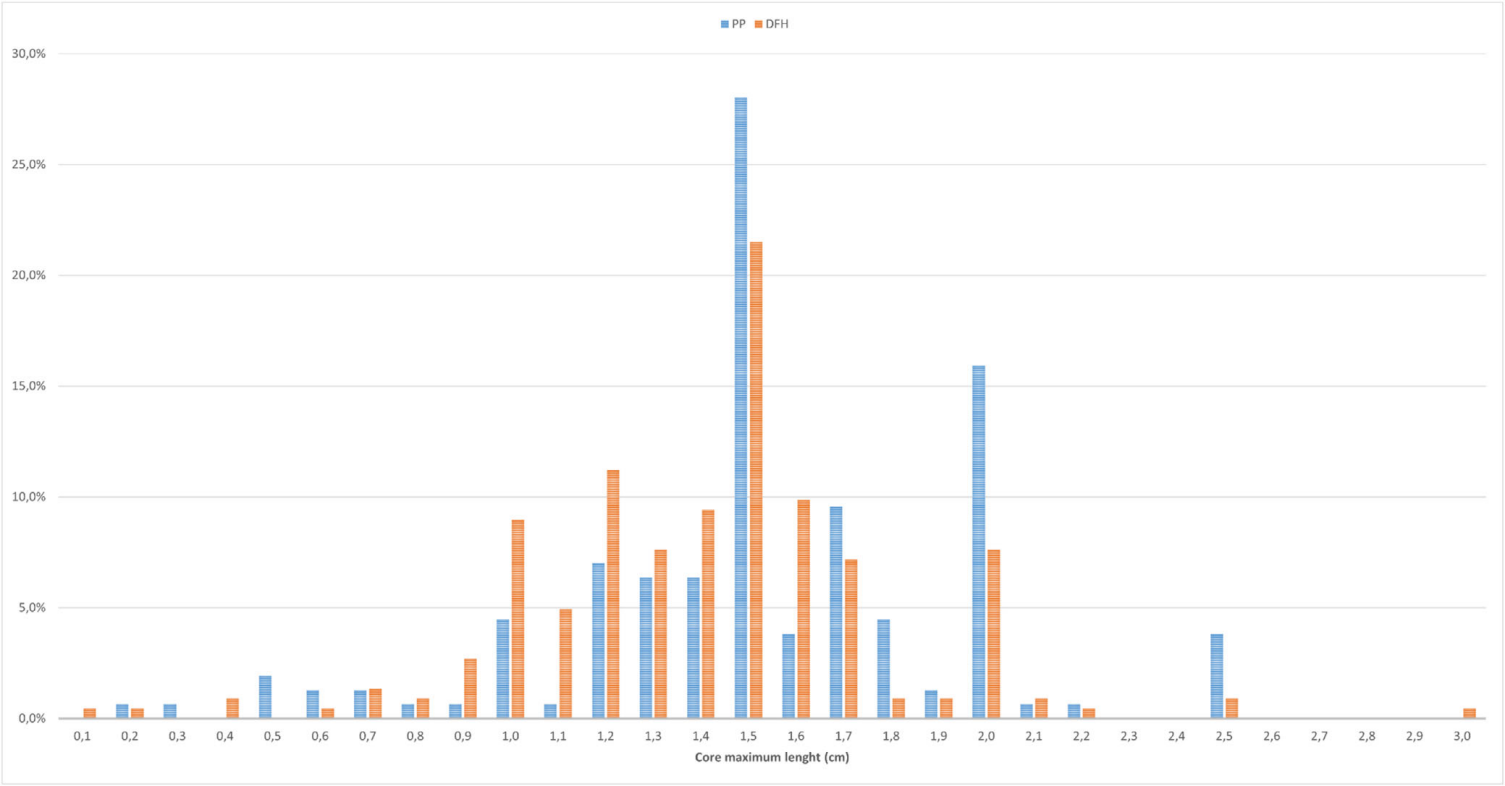
Observed rates for the variable ‘maximum diameter’ of cores taken from each multiparametric magnetic resonance imaging (mpMRI) target undergoing targeted biopsy with PrecisionPoint (PP) and double‐freehand (DFH).

The biopsy cores obtained from targets underwent detailed histological, dimensional and qualitative analysis (Table 4). No significant differences were observed between the two techniques regarding the average number of cores taken from each target stratified by PI‐RADS. An increasing number of cores were taken from lesions of larger diameter and higher radiological suspicion, with an average approaching five cores per target in lesions reported as PI‐RADS 4 or 5. A significantly higher number of cores positive for PCa was observed with PP compared to DFH (57.7% vs. 49.6%, *p* = 0.0002). This result was observed for each PI‐RADS score, reaching statistical significance in PI‐RADS 4 (50.7% vs. 44.4%, *p* = 0.04). The dimensional analysis of the cores did not reveal a significant difference in the minimum length of the material collected or the fragmentation rate. However, a statistically significant difference was observed in the maximum length of the cores, although they were distributed around the same median value, with PP at 1.50 cm (IQR 1.30; 1.80) versus 1.50 cm (IQR 1.20; 1.60) in DFH (*p* < 0.0001, Figure 4).

**TABLE 4:** Histological, dimensional and qualitative analysis of cores taken from multiparametric magnetic resonance imaging (mpMRI) targets.

	PP group targets (*n* = 166)	DFH group targets (*n* = 242)	*p*‐Value
Median (IQR), average (SD) or *n* (%)			
Average cores collected from mpMRI targets			
PI‐RADS 3	3.4	3.7	ns
PI‐RADS 4	4.7	4.7	ns
PI‐RADS 5	5.4	5.4	ns
Cores positive for PCa collected from mpMRI targets			
PI‐RADS 3	28 (28.3%)	40 (18.6%)	ns
PI‐RADS 4	240 (50.7%)	256 (44.4%)	0.04
PI‐RADS 5	258 (75.1%)	317 (71.2%)	ns
Total	532 (57.7%)	614 (49.6%)	0.0002
Core dimensional analysis			
Minimum length (cm)	0.60 (0.30; 1.00)	0.60 (0.40; 0.95)	ns
Maximum length (cm)	1.50 (1.30; 1.80)	1.50 (1.20; 1.60)	<0.0001
Core qualitative analysis			
0 fragments	100 (60.2%)	140 (57.9%)	ns
1 fragment	30 (18.1%)	37 (15.3%)	
≥2 fragments	27 (16.3%)	45 (18.6%)	
Not reported	9 (5.4%)	20 (8.3%)	

Abbreviations: DFH, double‐freehand; IQR, interquartile range; ns, not significant; PCa, prostate cancer; PP, PrecisionPoint.

### 3.4 Tolerability and complications

No deviations from the protocol regarding the administration technique of the anaesthetic were observed for either technique. An average volume of approximately 3.0 mL of anaesthetic per side was administered at the perineal plane in both PP and DFH techniques (Table 5). A significantly lower volume per side was administered in biopsies performed with the PP technique at the periprostatic tissues, with 4.3 (±2.0) mL compared to 5.9 (±1.9) mL in DFH (*p* < 0.001).

**TABLE 5:** Tolerability and complications.

	PP group (*n* = 154)	DFH group (*n* = 206)	*p*‐Value
Average (SD) or *n* (%)			
Anaesthetic administered per side (mL)			
Perineal plane	3.0 (±0.7)	3.0 (±0.4)	ns
Periprostatic plane	4.3 (±2.0)	5.9 (±1.9)	<0.0001
Reported VAS pain score	2.7 (±1.4)	2.7 (±1.6)	ns
Therapy with LMWH	11 (7.1%)	8 (3.9%)	ns
Monitoring of procedural complications			
Intra‐procedural vasovagal episode	2 (1.3%)	2 (0.9%)	ns
Post‐procedural vasovagal episode	0	5 (2.4%)	
Periprostatic haematoma	0	2 (0.9%)	
Clinically significant haematuria	0	0	
Acute urinary retention	0	1 (0.5%)	
Hospitalisation for post‐biopsy septicaemia	0	1 (0.5%)	

Abbreviations: DFH, double‐freehand; LMWH, low‐molecular‐weight heparin; ns, not significant; PP, PrecisionPoint; VAS, visual analogue scale.

The differences in the volume of local anaesthetic did not result in a significant difference in the average pain perceived by patients, which was 2.7/10.0 in both approaches. Monitoring of procedural complications revealed a low complication rate for both techniques, although a higher number was observed following biopsies performed with the DFH technique. All cases of vasovagal episodes occurred during outpatient biopsies and were successfully managed with saline infusion. All complications were classified as grade I according to the Clavien–Dindo classification, except for one case of hospitalisation for hyperpyrexia and urinary septicaemia in a high‐risk patient who received peri‐procedural antibiotic prophylaxis, which was classified as grade II.

## 4 DISCUSSION

This study has demonstrated the added value of the PrecisionPoint Transperineal Access System in cognitive prostate biopsy performed under local anaesthesia. The use of the device resulted in a higher rate of csPCa diagnoses from mpMRI targets compared to the DFH technique. The use of a perineal access cannula did not lead to an increase in reported pain or post‐biopsy complications, while also reducing the procedural requirement for anaesthetic.

The csPCa diagnosis rate using cognitive registration at our centre has proven to be similar to, if not better than, the rates described in multicentre randomised clinical trials that have established the role of mpMRI‐targeted biopsy or compared cognitive registration to MRI/TRUS software fusion. Specifically, the csPCa diagnosis rate in the PRECISION trial, derived from a combination of cognitive and fusion registration depending on the local protocols used in participating centres, was 39%, with 9% of ciPCa diagnosed and 44% of positive cores collected from targets.^23^ This phenomenon was also observed in comparison with the FUTURE and SmartTarget trials, designed to evaluate the superiority of software fusion over cognitive registration. In the FUTURE trial, the csPCa diagnosis rates for fusion, cognitive and MRI‐in bore registration were 27%, 26% and 25%, respectively, with ciPCa rates of 39%, 34% and 32% (*p* > 0.9).^24^ In the SmartTarget trial, the diagnostic performance of fusion versus cognitive registration was 54% and 53% for csPCa, 14% and 12% for ciPCa, respectively, with the best sampling efficiency achieved by taking the maximum number of cores allowed by the protocol (*n* = 3) from mpMRI targets.^25^ None of these studies demonstrated the superiority of MRI/TRUS software fusion registration over cognitive registration. This findings were confirmed in a recent meta‐analysis of randomised studies comparing the two registration methods exclusively for the transperineal approach. From the synthesis of evidence from eight eligible studies, the odds ratio (OR) for csPCa diagnosis between the two approaches was 1.01 (95% CI 0.74–1.37, *p* = 0.95).^26^ Similar results were provided by a 2024 multicentre analysis of TP‐Bx outcomes at three academic centres, with an OR of for csPCa detection of targeted biopsy cores of 1.01 (95% CI 0.55–1.84, *p* = 0.9).^27^ Consequently, the superior results obtained at our centre are not primarily attributable to the type of registration used. This study stratified the results by the visibility of mpMRI targets on TRUS, demonstrating a statistically significant difference in diagnostic performance in cognitive registration when the urologist identifies a hypoechoic area corresponding to the mpMRI‐described target. This finding corresponds to observations in a recently published retrospective analysis, which reported csPCa diagnosis rates of 56.3% for visible lesions and 10.5% for non‐visible lesions (*p* < 0.001). Notably, in multivariate analysis, target visibility on TRUS was associated with an OR for csPCa diagnosis in cognitive registration of 6.25 (95% CI 2.99–13.08, *p* < 0.001), whereas the OR for software fusion registration was 1.92 (95% CI 0.87–4.24, *p* = 0.107).^28^ These results, along with the quantitative synthesis of published evidence, support the hypothesis that the real added value of fusion registration biopsy might be in aiding the sampling of non‐TRUS‐visible targets. Therefore, the design of randomised trials aimed at confirming these findings is advocated.

It is of interest that in this analysis, the PP device ensured a higher csPCa diagnosis rate in PI‐RADS 4 targets even when not visible on TRUS. This finding reaches the lower limit of statistical significance and may be explained by qualitative parameters. Indeed, it is established that the optimisation of a prostate diagnostics service must involve the meticulous analysis of various quality checkpoints.^29^ In this regard, the PI‐QUAL 5/5 quality certification obtained by our centre confirms the high standard of imaging produced by the internal pathway. However, only half of MRI‐based biopsy procedures offered at our centre were informed by internal mpMRI scans. Therefore, the high diagnostic performance achieved in our study must primarily be influenced by aspects related to the biopsy technique. The implementation of a high number of cores collected from the target area in the biopsy strategy, with a distribution that considers the concept of *target penumbra*, may have significantly influenced the study results.^20^, ^21^ This hypothesis is supported by the high percentage of cores positive for PCa obtained in both DFH and PP techniques. A greater number of positive cores per sampled target are necessarily associated with a better characterisation of intra‐tumoral histological heterogeneity, resulting in a higher probability of sampling tissue from histologically aggressive disease foci, leading to a diagnosis of csPCa.^30^ Additionally, the statistically significant difference in diagnostic performance supporting the use of the PP cannula in transperineal prostate biopsy is also noteworthy. This phenomenon can be explained by the better quality of cores obtained using the perineal access system. It is known that the length of the sampled core is a fundamental quality indicator of biopsy sampling, with an OR for PCa diagnosis of 2.57 (95% CI 1.46–4.52) for cores longer than 11.9 mm.^31^, ^32^ The PP system technique, likely thanks to less needle distortion during biopsy compared to the DFH technique, ensured the collection of significantly longer cores. Consequently, the high level of diagnostic performance observed in this study may be explained by a combination of technical factors, including the adaptation of the biopsy strategy to recently published scientific evidence and the use of the perineal cannula. This is further supported by comparing the results obtained with PP at our centre with a quantitative synthesis of PP performance derived from the analysis of 16 publications. In the review, the cumulative diagnosis rate for csPCa detected was 42.6% (range: 14%–69.1%).^33^ Notably, a single study observed diagnosis rates higher than 55%, and the included studies also considered the percentage of csPCa diagnosed with systematic biopsies outside the target area, where the added value for csPCa diagnosis can reach an extra 9%–10%.^34^

The use of the PP system was also associated with high intra‐procedural tolerability. The VAS scores observed were in line with those described in the synthesis analysis (median range 2.8–4.5).^33^ The local anaesthesia protocol devised at our centre involved significantly reduced volumes of anaesthetics, with an average requirement of mepivacaine <200 mg per procedure, which is well below the safety limits. The VAS scores we observed are lower to those reported for both approaches in the PREVENT randomised trial as well as historical reports for the transrectal approach,^1^, ^35^ underscoring the importance of developing adequate expertise with anaesthesia protocols in TP‐Bx. The need for a smaller quantity of anaesthetic, despite using a transperineal metal cannula, can be explained by the ability to perform more controlled biopsy manoeuvres while keeping the needle in proximity to the deep anaesthesia sites with PP compared to DFH. The biopsy procedure under local anaesthesia proved to be overall safe with both techniques studied, and the complication rates were in line with those reported in the literature.^17^, ^33^, ^36^ This is noteworthy considering that all complications, except one, were classified as grade I according to Clavien–Dindo. The occurrence of a single episode of septicaemia in transperineal prostate biopsy in a patient who did not omit antibiotic prophylaxis further confirms the safety of the approach from an infection standpoint. The difference in complications between the two approaches was not significant, likely due to the paucity of such events. Continued prospective monitoring of results will help determine whether PP also offers an advantage in reducing post‐procedural complications compared to DFH.

This analysis of the results from a prospective audit is not devoid of limitations. The allocation of patients to receive biopsies using PP versus DFH was determined by the judgement of an experienced tutor based on the training urologist's experience at the time of the procedure. Therefore, these results require confirmation in a randomised trial, which is scheduled to start at our centre. Due to the limited duration of the prospective audit period, this analysis cannot assess the impact of continued introduction of stringent criteria regarding the execution and quality of pre‐biopsy mpMRI. As demonstrated in a recently published dynamic data‐based analysis, the effect of introducing mpMRI in a prostate diagnostics service is progressive over the years.^37^ Thus, part of the results obtained at our centre could be attributed to these effects. However, it should be noted that the allocation to PP and DFH has varied regularly since the introduction of PP in May 2021, so this limitation is considered minor. The visibility of mpMRI targets on TRUS is a highly subjective parameter, although crucial for performing a biopsy sampling in cognitive registration. Nevertheless, the organisation of the prostate biopsy service at our centre ensures that ultrasound images are evaluated by at least three individuals in each phase: an expert during the planning TRUS, a tutor during the biopsy procedure and the training urologist. The involvement of three urologists, including two experts in interpreting TRUS images, limited the bias associated with subjectivity.

## 5 CONCLUSIONS

The results of this study confirm the high diagnostic performance and safety of transperineal prostate biopsy under local anaesthesia. The use of the PrecisionPoint Transperineal Access System has enabled more precise and higher quality biopsies, resulting in improved histological characterisation of prostate cancer. The perineal cannula was also associated with a reduced need for local anaesthetic and a lower number of complications, none of which were classified as severe. These findings suggest that probe‐tethered access cannulas may become the standard of care in transperineal prostate biopsy. The design of randomised clinical trials focused on the role of the perineal access cannulas in prostate biopsy is encouraged, with a specific emphasis on the devices' role in various image registration techniques, accounting for the visibility of mpMRI targets in transrectal prostate ultrasound.

## AUTHOR CONTRIBUTIONS

*Conceptualization*: Luca Orecchia and Roberto Miano. *Methodology*: Luca Orecchia, Roberto Miano and Stefano Germani. *Formal analysis*: Luca Orecchia and Roberto Miano. *Investigation*: Luca Orecchia, Stefano Germani, Gaia Colalillo, Angelica Fasano, Matteo Ricci, Eleonora Rosato and Guglielmo Manenti. *Writing—original draft*: Luca Orecchia. *Writing—review and editing*: Roberto Miano, Simone Albisinni, Guglielmo Manenti and Enrico Finazzi Agrò. Supervision Roberto Miano.

## CONFLICT OF INTEREST STATEMENT

The authors have no financial or non‐financial interests related to this work to declare. No funding was received for conducting this study.
